# Biocultural determinants of overweight-obesity among adult women experiencing the nutritional transition in the Democratic Republic of Congo

**DOI:** 10.3389/fnut.2024.1341710

**Published:** 2024-11-25

**Authors:** Wakenge Wakilongo, Souheila Abbeddou, Lucie Vanhoutte, Norbert Amougou, Muko Mubagwa, Clémence Elmira, Patrick Pasquet, Emmanuel Cohen

**Affiliations:** ^1^Unité Mixte de Recherche (UMR) 7206, Eco-anthropologie (EA), Museum National d'Histoire Naturelle, Centre National de la Recherche Scientifique (CNRS), Université Paris Cité, Paris, France; ^2^Département de Nutrition, Centre de Recherche en Sciences Naturelles (CRSN), Lwiro, South Kivu, Democratic Republic of Congo; ^3^Department of Public Health and Primary Care, Ghent University, Ghent, Belgium

**Keywords:** overweight-obesity, determinants, nutritional transition, women, Democratic Republic of Congo

## Abstract

**Introduction:**

The African Great Lakes region is experiencing rapid urbanization, which is leading to a nutritional transition and its related chronic diseases. Similar to other Great Lakes countries, the nutritional transition in the Democratic Republic of Congo (DRC) is reflected by increased non-communicable diseases, including morbid obesity. The 2014 Demographic Health Survey (DHS) revealed a rising incidence of overweight among women, ranging from 10% in 2001 to 16% in 2014. Furthermore, over 20% of individuals in several provinces of the DRC are classified as overweight-obese. This study aimed to investigate the prevalence as well as the main biocultural determinants of overweight-obesity among adult women in the DRC.

**Methods:**

In a cross-sectional survey, including a representative sample of adult Congolese women living in the South Kivu province, participants were randomly recruited using a two-stage cluster sampling technique after an initial urban–rural stratification. The estimation of the was based on previous results from DHS. Thus, a total of 495 individuals were selected, including 325 urban and 170 rural subjects. Data were collected from households by dietitians who were specifically trained for this survey.

**Results:**

In this study population, the prevalence of overweight-obesity was 33.6%, with 7.1% classified as obese. The prevalence of obesity was significantly higher among urban people, while all subjects with obesity were from the older age group (>35 years). Using binomial logistic regressions, it was observed that overweight-obesity had a significant positive correlation with the duration of urban residence, namely, **Migrants' status:** “New residents” 4.6 [1.9–11.7] *p* < 0.003, “Long term residents” 8.7 [3.5–21.5] *p* < 0.001; **Socio-economic status (SES):** “High” 2.4 [1.1–5.3] *p* < 0.03, and **Stoutness valorization:** “Yes” 6.1 [3.4–10.9] *p* < 0.001. In a pathway analysis conducted based on a structural equation model (SEM), we discovered that urban residence and SES were associated with an increase in overweight-obesity, with a positive correlation with processed food consumption and a negative correlation with physical activity. Age was associated with an increase in overweight-obesity through a negative association with physical activity, whereas stoutness valorization directly increased overweight-obesity.

**Discussion:**

In order to properly guide public health policies, public authorities in the DRC should consider the main findings of this original study, which identify how socio-demographic and socio-ecological factors contribute jointly to the rising prevalence of overweight-obesity in the country.

## 1 Introduction

Obesity is a risk factor for serious health problems (1), especially chronic diseases, which can highly impair the quality of life. However, the persistent rise in overweight-obesity rates is alarming worldwide. Adults' who are overweight or obese are defined by their body weight (kg) and height (m) squared ratio (body mass index, BMI), from which they are derived. In 1990, it was estimated that around 12% of the world's population was obese, with the rate reaching 20% in 2015, with a higher increase among women (2). In 2017, the World Health Organization (WHO) estimated that 1.9 billion adults worldwide were classified as overweight-obese, with 650 million of them being obese (2). By 2030, 1 billion people will be classified as obese, representing one in five women and one in seven men (3). Previously considered diseases of rich countries, overweight-obesity is now increasing in low-income countries. Indeed, they are currently undergoing a rapid urbanization process, characterized by increased access to energy-dense processed foods and motorized transports. This is favoring both overnutrition and sedentary behaviors that lead to an obesogenic nutritional transition (4, 5). During the last decades, the prevalence of obesity has increased significantly in Africa, from 5%−10% globally during the 1990s to a maximum of 30%−50% actually in some regions. The highest prevalence is in urban areas (6).

Few studies have been conducted on the rising obesogenic environment in the Democratic Republic of Congo (DRC) (7–9) in the context of nutritional transition. Some studies have reported an increasing incidence of cardiometabolic diseases (10, 11). Hence, the latest Demographic Health Survey (DHS) revealed that the proportion of women who have overweight, or women with overweight has increased from 10% in 2001 to 11% in 2007, and 16% in 2014, with over 20% of individuals in several provinces (Kinshasa, North Kivu, and South Kivu) exhibiting overweight-obesity (7, 12). A recent study conducted among adult women in Kinshasa in 2023 confirmed this trend, with an overweight-obesity prevalence of 32.7% (9). Musung et al. have also demonstrated a significantly higher mean BMI in adolescent girls from Lubumbashi compared to their male counterparts, with potential poor cardiometabolic consequences (8).

The rapid urbanization dynamic experienced by African countries leads to an obesogenic lifestyle, including jointly a higher energy-dense food accessibility (13, 14), increasing sedentary behaviors (5, 15), and a persisting sociocultural valorization of stoutness (6, 16). These biocultural risk factors are primarily responsible for an imbalance between energy intake and expenditure, leading to overweight-obesity and related chronic diseases. In the context of increasing urbanization, African cities attract rural populations looking for a potential better life, abundance, and comfort with more work opportunities and easier access to basic services (17). Therefore, urbanization, which represents the major environmental dimension of the nutritional transition, is shaped by multiple socio-demographic and socio-ecological factors (5), leading together to obesity and related diseases in Africa (18), especially among women (17, 19).

Bukavu, the capital city of the South Kivu province in the east of the DRC (20), is an illustrative example of the rapidly growing African cities due to the recent rural exodus and the massive displacement of populations (21). Furthermore, the rapid transformation of Bukavu is exacerbated by the social, political, and economic instability and the recurrent local wars taking place in the surrounding region (20, 21). Despite this rapid urban transition in South Kivu, little is known about the health impact of the intrinsic nutritional transition within this environmental dynamic urban transition (8, 9). In addition, a thorough investigation of the risk factors for overweight-obesity has never been conducted in this transitional country. Since previous research indicated that women are more susceptible to overweight-obesity than men, particularly during the initial nutritional transition stages (17, 19), this original rural–urban study conducted in South Kivu province aimed to focus on the biocultural determinants that contribute to overweight-obesity among adult women, who are in the first at-risk group to develop morbid obesity (14).

From March to June 2017, we conducted an innovative survey among adult women from Bukavu and its surrounding rural area (Katana area at the northeast of South Kivu province) in order to assess the prevalence of overweight-obesity and explore its main biocultural determinants (body image, physical activity, and dietary intake) in the context of the ongoing nutritional transition in the DRC.

## 2 Materials and methods

### 2.1. Study design and sampling

A sample size was established by considering 26.5% of the expected prevalence of overweight-obesity, 95 % of the confidence level, 3% of desired precision, and 5% of risk α. These statistical criteria were defined from the previous DHS conducted in the DRC in 2007 and 2014 (7, 12). This cross-sectional study was conducted using a multi-stage clustering sample (22), which was also used in the DHS of the DRC. To avoid potential dropout during the study, the sample size has been increased by 10%. To obtain a representative sample that encompasses the diversity of the study population in South Kivu province, the subjects' recruitment was based on the identification of the strata (i.e., rural–urban residence) and a two-stage random sampling of clusters within this strata. During stage 1, 30 representative neighborhoods in the urban and 20 representative villages in the rural areas were selected. In stage 2, the primary responsible mother in each household selected was recruited (< 5% of all households). Finally, 495 participants (325 in urban and 170 in rural areas) with complete and reliable data were selected (Supplementary Figure S1). The rural–urban proportion of the sample and selected rural–urban areas were determined from provincial population registers in order to be representative of the target populations. These registers, which are used in population surveys such as DHS (7, 12), are based on regional censuses commissioned by the DRC government and constructed through rigorous and regionally representative sampling (7). Subjects were included in the study if they were: (i) female, (ii) at least 18 years old, (iii) not reportedly pregnant, (iv) and did not have a disability or any other chronic or infectious pathologies likely to affect corpulence. These last two exclusion criteria are particularly important for the anthropometric results.

### 2.2 Data collection

From March to June 2017, we conducted a quantitative survey using a global questionnaire comprising eight headings: identification of the study subjects, socio-demographic characteristics, socio-economic status (SES), migratory history, dietary intake, physical activity, body perception, and anthropometric measurements. WE used specific survey tools, from previously validated instruments (23–26), to administer questionnaires assessing SES, 24-h qualitative dietary intake, food frequency, physical activity, body weight perceptions, and an anthropometric measurement protocol. The survey was based on face-to-face interviews conducted within the household in a separate space, for confidentiality, by Congolese dieticians (in French or Swahili vernacular language when necessary) trained to administer the study protocol.

#### 2.2.1 Socio-demographic characteristics

***Age*** was determined from an identity document or as reported by the participant and was classified into 3 categories: 18–25 years old; 26–35 years old; and >35 years old, based on the terciles of its distribution in the study population. ***Marital status*** was categorized into “married” and “unmarried (with divorce, single, widow, and mistress).” Parity was not collected because it is strongly correlated with age and is associated with marital status, particularly in the DRC, where motherhood is expected with age and marital life. ***Place of residence*** was categorized as “urban” for an urban residence for more than 1 year and “rural” for a shorter duration. This categorization allowed to balance the rural–urban stratification since our sample included urban dwellers for less than a year. The ***ethnicity*** of the participants primarily comprised two major ethnic groups in the DRC, namely *Shi* and *Lega* (27). ***Urban duration*** was calculated from the length of residence in an urban area and was classified as short duration if the duration was < 15 years and long duration if the duration was ≥15 years. The ***SES*** was constructed from the goods owned by families, considered as a proxy of wealth through the first axis of a principal component analysis (PCA) summarizing the variability of household assets (44.5% of total inertia, Supplementary Figure S2), as described by Kombiané (23), and updated by Escofier and Pagès (28). Scores of SES status were categorized into terciles in order to define low, medium, and high SES levels. A Kaiser–Mayer–Olkin (KMO) index of 0.727 and a highly significant Bartlett test of sphericity (*p* < 0.0001) indicated both strong sampling adequacy and a correlation matrix.

#### 2.2.2 Socio-ecological characteristics

##### 2.2.2.1 Stoutness valorization

In order to accurately assess body weight perceptions and identify the social valorization of overweight-obesity, the Body Size Scale (BSS) was used. This validated tool presents real human photos of body size for both sexes, coded from 1 to 9, and covers the total BMI gradient from underweight to obesity (25). A body image assessment guide (BIAG) was created to compare body weight local norms with scientific norms measured with the BSS. The BIAG is composed of two questions on Perceived Body Size (PBS) and Ideal Body Size (IBS) for the participant and one's partner. The degree of stoutness valorization was determined by comparing the IBS score with a threshold established according to the silhouettes' BMI on the scale, as follows: no valorization of stoutness below the 5^th^ silhouette (beginning of overweight on the BSS), and a valorization of stoutness above the 5^th^ silhouette (15).

##### 2.2.2.2 Physical activity

The International Physical Activity Questionnaire (IPA) was used to assess the level of physical activity (26). It calculates the equivalent metabolic task (MET/minutes) of each physical activity (intense, moderate, walking, and sedentary behaviors) according to its respective duration and number of practiced days per week. A score of physical activity, expressed in MET/minute/week, was calculated after considering all the activities declared by the participant during the last 7 days preceding the interview. This physical activity score was also categorized into three terciles: [0–0.8]; [0.9–2.2]; and [2.3–4.9] for low, medium, and high physical activity levels, respectively.

##### 2.2.2.3 Dietary intake

###### 2.2.2.3.1 Dietary diversity score (DDS)

A qualitative 24-h dietary recall, developed by the Food and Agriculture Organization (FAO) (29), was used to assess the diversity of food groups consumed by participants. It captures the intake of 16 main food groups, including cereal, white roots and tubers, vitamin A-rich vegetables and tubers, dark green leafy vegetables, other vegetables, vitamin A-rich fruits, other fruits, organs, flesh meats, eggs, fish, legumes and nuts, milk and dairy products, oils and fats, sweets, and spices and beverages. Based on the median, dietary diversity was categorized as high diversity if the participant's dietary diversity score was at least 7, and low diversity if the score was lower than 7.

###### 2.2.2.3.2 Food consumption frequency

Using a culturally relevant food frequency questionnaire (FFQ) of 80 food items (including soft and alcoholic drinks) divided into 28 food groups (30), we collected information about the consumption frequency of each food item during the previous 7 days. The results from the seven-day FFQ were then analyzed using a PCA. Food intake was classified as “processed” vs. “traditional” from a specific anthropological literature of the DRC, which characterizes foods from historical culinary practices and processed foods from industrialization (31). Our PCA results, based on the foods having the highest contribution on the two first factors, revealed that the foods significantly associated (>0.3) with the first factor correspond to the “processed” food category, and the foods significantly associated (>0.3) with the second factor correspond to the “traditional” food category (32–34). Therefore, the two first factors were kept, representing, respectively, “processed foods” and “traditional foods” (Supplementary Figure S3, Supplementary Table S1). We used the KMO index (0.7) and the Bartlett's sphericity test (*p* < 0.001) to confirm both the strength of sampling adequacy and the correlation matrix of the PCA. The food consumption frequency scores were then aggregated into deciles, with “High” coded by (+) and “Low” coded by (–) to carry out a multiple correspondence analysis (MCA) aiming to determine a dietary consumption pattern according to urban duration. Both D1 and D2 (32.1% total inertia) of the MCA synthesized the maximum amount of information to describe the food consumption and depict it graphically.

It is noteworthy that tobacco consumption was not collected in this study because tobacco is rarely used in the DRC, particularly among women (7).

#### 2.2.3 Anthropometric measurements

The participant's weight in kilograms was measured standing with minimal clothing using an electronic scale (UNICEF SCALE) with a capacity of 150 kg and a precision of 100 g. The height was measured standing without shoes, using a portable stadiometer (Sieber Hegner, Zurich, Switzerland), with an accuracy of 1 mm. The BMI was used to assess the nutritional status of participants, expressed in Kg/m^2^. The BMI ranges were defined according to the WHO thresholds applicable to adults (35): underweight: < 18.5 Kg/m^2^; normal weight: (18.5–24.9) Kg/m^2^; overweight: (25.0–29.9) Kg/m^2^; and obesity: ≥0.0 Kg/m^2^. Study subjects were classified based on whether they had overweight-obesity (BMI ≥ 25 kg/m^2^) or not (BMI < 25 Kg/m^2^).

### 2.3 Data analysis

Additional tables and figures regarding score construction are provided in the Supplementary material. Simple linear regressions were employed to evaluate distinct associations between physical activity, frequency of processed food consumption (PFC), BMI, and urban duration. The comparison of variables and statistical tests necessitated a weighted coefficient based on the differential rural–urban clustering of the study (with a coefficient of 1.4 and 0.2 for the rural and urban areas, respectively) and an adjustment for age. Chi-squared/exact Fisher tests were used for categorical variables, and the Wilcoxon rank test was used for continuous variables.

The MCA was carried out to assess the relationships between food consumption frequency and urban duration. The MCA graph was used to display consumption food patterns according to urban duration. To investigate the biocultural factors associated with overweight-obesity, we performed an adjusted binomial logistic regression. The adjusted odds ratios (OR), their 95% Confidence Intervals (95% CI), as well as the degree of significance (*p* < 0.05) were analyzed to identify the factors that are independently associated with overweight-obesity. Finally, a structural equation model (SEM) was used to determine the casual pathways between socio-demographic factors (age, ethnicity, marital status, urban duration, and SES), socio-ecological factors (dietary intake, physical activity, and stoutness valorization), and overweight-obesity. The software R (v.4.2.1) and R Studio (v.2022.07.1) were used for data analysis, whereas XLSTAT software (from Excel) was used to conduct factorial analyses (MCA and PCA) and reduce the substantial variance into proxies.

### 2.4 Ethics

The study was conducted in accordance with the South Kivu provincial ethics committee (UCB/CIES/NC/015/2024). After being informed about the objective of the study, oral approval and written consent were obtained from each participant before the start of the study.

## 3 Results

### 3.1 Socio-demographic characteristics

Our findings (Table 1) indicate that the mean age of urban dwellers was not significantly different from that of rural dwellers. However, nearly half of urban subjects were over 35 years old, compared to only 10% of rural dwellers. Among urban dwellers, over half have lived in the urban area for < 15 years. A third of urban dwellers were in the highest SES category, compared to < 10% of their rural counterparts. Majority of the participants were from the Shi ethnicity.

**Table 1:** Socio-demographic characteristics of the study population according to living area.

**Characteristics**	**Overall *N =* 495**	**Rural *N =* 241**	**Urban *N =* 254**	**p**
**Age class**				**0.048**
18–25 years	36.0%	42.8%	29.5%	
26–35 years	35.5%	47.7%	24.1%	
>35 years	28.5%	9.5%	46.4%	
**Age (means)**	30 ± 9	27 ± 7	33 ± 10	0.1
**Migrant status**				**< 0.001**
Rural	48.6%	100.0%	_	
New residents (< 15 years)	27.1%	_	52.6%	
Former residents (≥15 years)	24.4%	_	47.4%	
**SES**				**0.003**
Low	23.8%	21.6%	25.9%	
Medium	54.9%	71.6%	39.1%	
High	21.3%	6.8%	35 %	
**SES score (means)**	−0.08 ± 0.87	−0.34 ± 0.35	0.17 ± 1.1	0.2
**Marital status**				0.5
Not married	24%	22.2%	25.7%	
Married	76%	77.8%	74.3%	
**Ethnicity**				0.7
Lega	19.6%	18.4%	20.8%	
Shi	80.4%	81.6%	79.2%	

*p* < 0.05 in bold.

### 3.2 Prevalence of overweight-obesity

The prevalence of overweight-obesity in the study sample was 33.6%, including 7.1% of participants who were obese. The overweight prevalence was significantly higher among urban dwellers, with 49.1%, compared to rural dwellers with a prevalence of 17.2%. Similarly, obesity prevalence was significantly higher among urban dwellers (11.5%) as compared to a prevalence of 2.5% among rural dwellers (*p* < 0.001). Overweight-obesity prevalence did not exhibit significant a correlation with age. The prevalence was 34.5% among the age group 18–25 years, 28.9% among the age group 26–35 years, and 36.6% among the age group older than 35 years. All subjects (100%) with obesity were within the oldest group.

### 3.3 Socio-ecological characteristics

#### 3.3.1 Stoutness valorization

**Figure 1:**
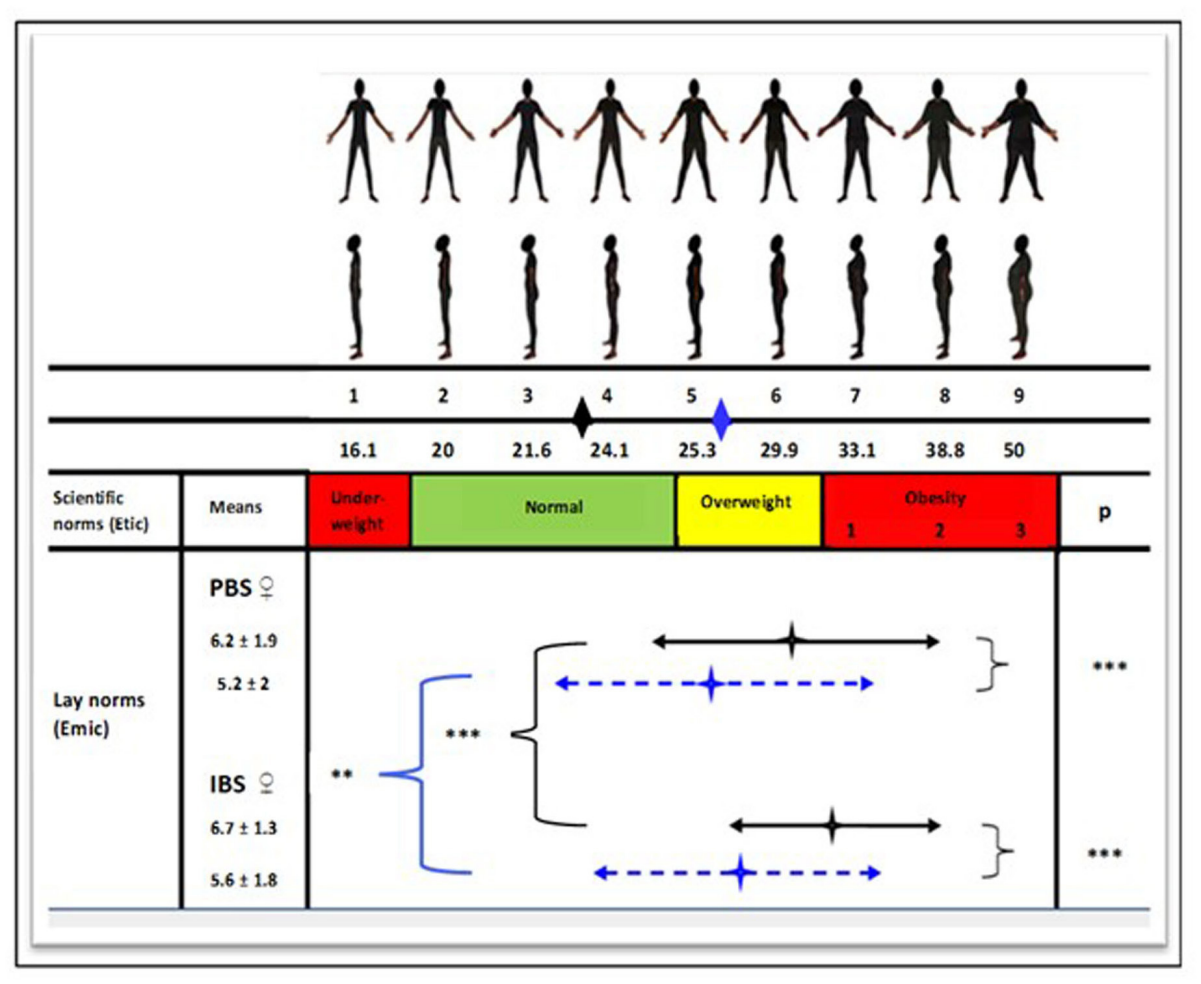
Perception of body image in women by area of residence. The marks in the middle of the two-way arrows correspond to the mean BMIs as perceived by women. The two-way arrows correspond to the standard deviation. The black and blue diamonds just below the body silhouettes indicate BMI averages for rural and urban populations, respectively. A *t-*test was performed between the two samples: **p < 0.01; ***p < 0.001. 
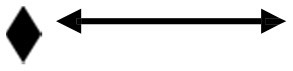
, Rural area; 
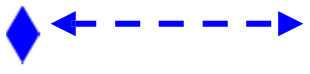
, Urban area. PBS, Perceived body size; IBS, Ideal body size.

Our results indicate that rural dwellers have a larger perceived body size (PBS) than urban dwellers (Figure 1). Many rural dwellers underestimated their own weight. The diamonds in the middle of the bidirectional arrows, representing respectively means and standard deviations (SD), indicate that rural dwellers' PBS was situated in the overweight category, while their real BMI mean was situated in the normal weight category. Both rural and urban dwellers had IBS means that were situated in the overweight category. However, the SD for rural dwellers covered the overweight and obesity categories, while some urban participants had an IBS within the normal weight category. Furthermore, we noted that IBS scores were higher than PBS scores in both groups, with a more accentuated tendency in rural areas.

#### 3.3.2 Dietary intake

Our results presented in Table 2 showed a high dietary diversity score (DDS). Almost two-thirds of participants consumed at least seven food groups during the previous 24 h. In addition, there was no significant difference in DDS between both areas of residence. Moreover, our results showed that processed foods were more consumed by urban dwellers compared to rural dwellers, while traditional foods were more consumed in rural areas compared to urban areas.

**Table 2:** Socio-ecological characteristics according to living area.

**Characteristics**	**Overall *N =* 495**	**Rural *N =* 241**	**Urban *N =* 254**	***p***
**Physical activity level**				**< 0.001**
Low	69.4%	45.5%	92.1%	
High	30.6%	54.5%	7.9%	
**Physical activity score (means)**	1.2 ± 1.1	1.7 ± 1.3	0.7 ± 0.6	**< 0.001**
**Stoutness valorization**				**< 0.001**
No (≤ 4)	27.7%	18.2%	36.7%	
Yes (>4)	72.3%	81.8%	63.3%	
**Dietary Diversity Score (DDS)**				0.8
Low (< 7)	38.7%	39.9%	37.5%	
High (≥7)	61.3%	60.1%	62.5%	
**DDS (means)**	6.9 ± 1.7	6.7 ± 1.4	7 ± 1.8	0.5
**Processed food consumption**				**< 0.001**
Low	83%	97.8%	69.1%	
High	17%	2.2%	30.9%	
**Processed food score (means)**	−0.2 ± 0.9	−0.9 ± 0.7	0.4 ± 0.8	**< 0.001**
**Traditional food consumption**				**< 0.001**
Low	81%	69%	92.3%	
High	19%	31%	7.7%	
**Traditional food score (means)**	0.1 ± 1	0.4 ± 1	−0.3 ± 0.9	**< 0.001**

*p* < 0.05 in bold.

**Figure 2:**
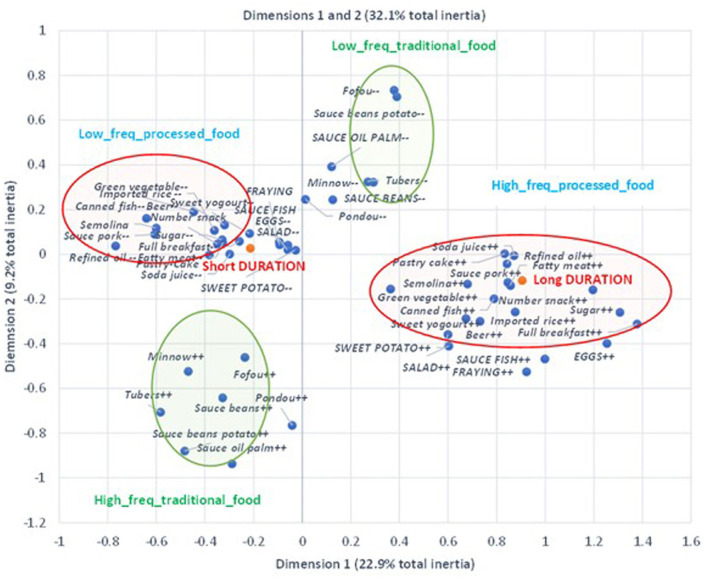
Consumption food pattern with urban duration. Distribution of very regularly consumed food according to urban duration: the additional modalities in red color represent the variable “Urban duration,” whose modalities “Short duration” indicate residence in a rural environment or urban duration <15 years, and “Long duration” indicates urban duration more than 15 years. The variables in red circles (followed by + and – signs) indicate, respectively, the very frequent vs. not frequent consumption of ultra-processed food. The variables in green circles (followed by + and – signs) indicate, respectively, the very frequent vs. not frequent consumption of traditional foods. The ellipses were plotted according to factor loadings >0.3 for both Dimensions 1 and 2. All variables were significantly correlated with either Dimension 1 or 2.

Figure 2 shows that, in an urban setting, processed foods were more frequently consumed by individuals with an urban duration of ≥15 years, while traditional foods were more regularly consumed by individuals with a shorter urban duration.

#### 3.3.3 Physical activity

Physical activity was generally low in the study population, with two-thirds of the study sample reporting low physical activity (Table 2). Physical activity was found to be significantly higher among rural dwellers as compared to their urban counterparts.

#### 3.3.4 Relationships between BMI, PFC, physical activity, and urban duration

**Figure 3:**
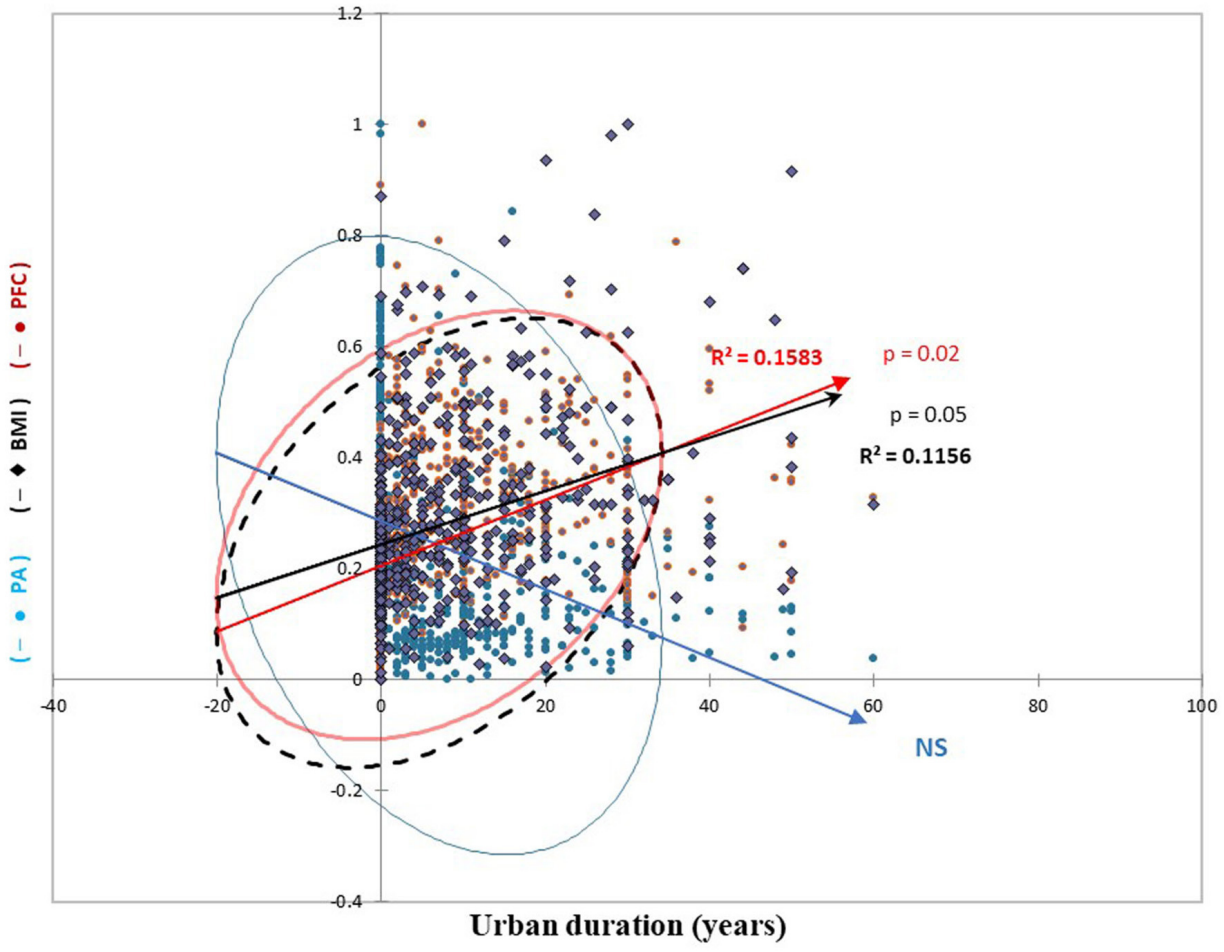
Physical activity (PA), body mass index (BMI), and processed food consumption (PFC) according to urban duration. The y-axis data has been standardized for comparability. The circles synthesize the point clouds to calculate the different regression lines. NS, Non-Significant.

Figure 3 shows that PFC frequency and BMI were positively associated with urban duration, whereas physical activity was negatively associated with urban duration.

### 3.4 Predictors of overweight-obesity

#### 3.4.1 Overweight-obesity-adjusted and unadjusted factors

The results from the logistic regression as presented in Table 3 show that urban duration, SES, and stoutness valorization were independently and positively associated with overweight-obesity. Urban dwellers, independently of urban duration, were more at risk of having overweight-obesity compared to rural dwellers. Similarly, participants with the highest SES were more at risk of having overweight-obesity than those within the lowest SES tercile. Finally, participants who valorized stoutness were more at risk of having overweight-obesity than those who did not valorize stoutness.

**Table 3:** Odds ratio (OR) and 95% confidence intervals (CI) between subjects classified and non-classified as overweight-obese, non-adjusted and adjusted by binomial logistic regression.

	**OR-non-adjusted for overweight-obesity**		**OR- adjustedfor overweight-obesity**	
**Characteristics**	**OR 95% CI**	***p***	**OR 95% CI**	***p***
**(Intercept)**			0.03 [0.01–0.19]	**< 0.001**
**Age (means)**	1.01 [0.98–1.05]	0.36	1.00 [0.97–1.03]	0.9
**Marital status**				
Non-married	1—		1—	
Married	1.16 [0.69–1.92]	0.57	1.27 [0.70–2.33]	0.4
**Ethnicity**				
Lega	1 —		1—	
Shi	0.90 [0.45–1.79]	0.75	1.02 [0.49–2.1]	>0.9
**Urban duration**				
Rural	1 —		1 —	
New residents	3.45 [1.76–6.78]	**< 0.001**	4.64 [1.85–11.7]	**0.003**
Long term residents	6.65 [3.47–12.7]	**< 0.001**	8.72 [3.54–21.5]	**< 0.001**
**SES**				
Low	1 —		1 —	
Medium	1.06 [0.57–1.98]	0.85	1.51 [0.71–3.19]	0.3
High	3.45 [1.60–7.44]	**0.003**	2.39 [1.08–5.30]	**0.034**
**Physical activity (means)**	0.64 [0.50–0.80]	**< 0.001**	0.84 [0.63–1.13]	0.2
**Stoutness valorization**				
No	1 —		1 —	
Yes	3.36 [2.03–5.58]	**< 0.001**	6.10 [3.43–10.9]	**< 0.001**
**Dietary diversity score**				
Low	1 —		1 —	
High	0.94 [0.56–1.59]	0.82	0.85 [0.51–1.41]	0.5
**Processed food consumption**				
Low	1 —		1 —	
High	1.38 [0.86–2.21]	0.18	0.73 [0.45–1.20]	0.2

The lines (-) indicate the modalities taken as reference, *p* < 0.05 in bold.

#### 3.4.2 Driver pathway analysis

**Figure 4:**
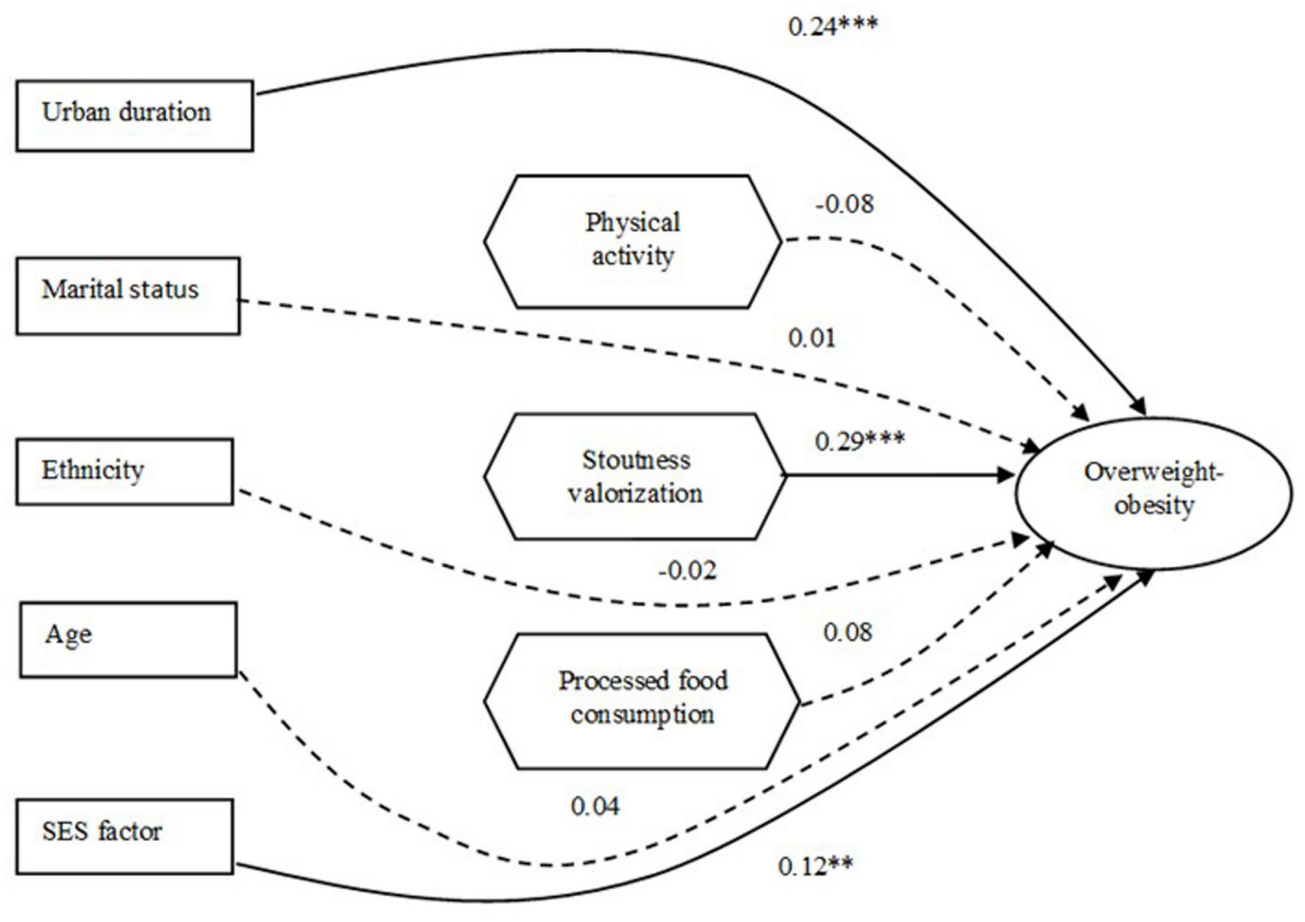
Results of structural equation modeling (SEM) show direct associations between socio-demographic/socio-ecological factors and overweight-obesity status. Bold, solid lines show significant associations, and dotted lines show non-significant associations. Their coefficients and their degrees of significance are indicated by numbers and stars: **p < 0.01; ***p < 0.001.

**Figure 5:**
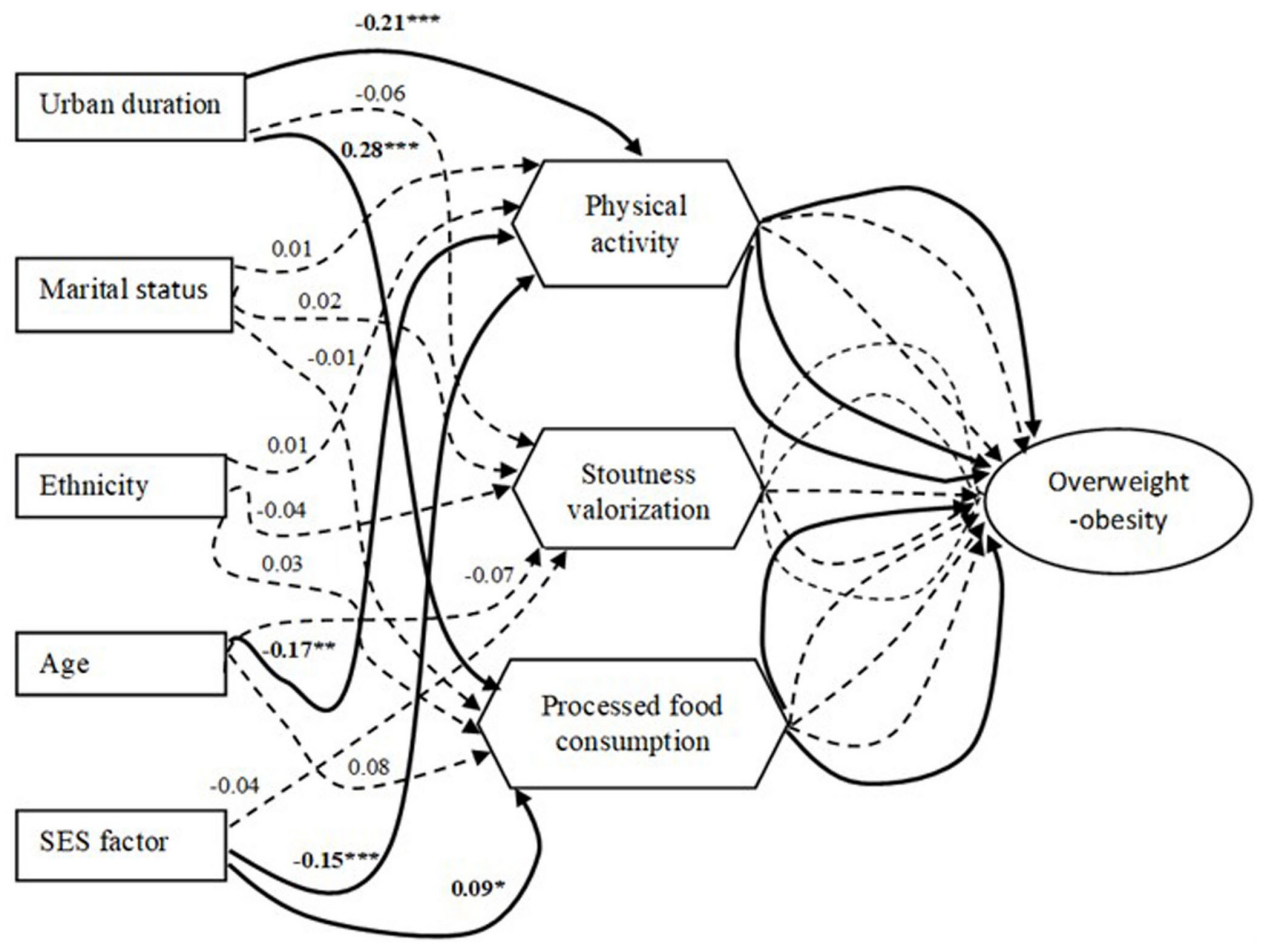
Results of structural equation modeling (SEM) show indirect associations between socio-demographic/socio-ecological factors and overweight-obesity status. Bold, solid lines show significant associations, and dotted lines show non-significant associations. Their coefficients and their degrees of significance are indicated by numbers and stars: *p < 0.05; **p < 0.01; ***p < 0.001.

The results of the SEM presented in Figures 4, 5 on the relationships between socio-demographic and socio-ecological factors and overweight/obesity show direct and indirect driver pathways.

##### 3.4.2.1 Direct pathway association

Urban duration, SES, and stoutness valorization were positively associated with overweight-obesity.

##### 3.4.2.2 Indirect pathway association

Some socio-demographic factors influenced overweight-obesity through intermediate socio-ecological factors. Urban duration and SES were positively associated with overweight-obesity, mediated positively by PFC and negatively by physical activity. Age was also found to be positively associated with overweight-obesity through a negative mediation with physical activity.

## 4 Discussion

The prevalence of overweight-obesity was high in this study sample of Congolese women. The socio-ecological factors analyzed showed that physical activity was significantly higher in rural areas than in urban areas and that PFC was significantly higher in urban areas than in rural areas, with a reversed trend for traditional food consumption frequency. In addition, the stoutness valorization was significantly higher in the rural area. It was demonstrated that urban duration, SES, and stoutness valorization were directly associated with overweight-obesity, while physical activity and PFC mediated the associations between overweight-obesity and urban duration, SES, and age (only physical activity for the last one).

Our results indicate that overweight-obesity is a public health issue in this region of the DRC, especially in urban area of South Kivu. The elevated prevalence of 33.6% observed could be attributed to the increasing urbanization of Bukavu, resulting from the ongoing and actual rural exodus experienced by this expanding city. The increasing urban environment is shaped by the globalization of the market economy which creates an obesogenic socio-ecological environment that exposes the population to overweight-obesity, as observed in Cameroon and Burkina Faso (16, 24). These results are in line with the DHS reporting in 2014 a prevalence of overweight-obesity of 26.5% and 27.8% in South and North Kivu, respectively, and a prevalence of 32.2% in Kinshasa, the capital city of the country (7). A similar prevalence was reported in Uganda (36), while the prevalence of overweight-obesity was higher in Cameroon, Tanzania, Nigeria, and South Africa, probably due to their advanced nutritional transition stages (16, 36).

In this study population, several socio-demographic factors interacted with socio-ecological factors leading to overweight-obesity, as observed in Cameroon by Cohen et al. (16). Our study findings indicate that urban duration and higher SES are significant risk factors for overweight-obesity, mediated by a decrease in physical activity and an increase in PFC. Similar trends are discussed in other studies conducted by Cohen et al. (15) in Senegal; Popkin et al. (5); and Delisle (17) in low and middle-income countries, especially in Benin and Burkina Faso. The improving SES has also been identified as a risk factor for overweight-obesity in other studies conducted in France and Cameroon (16, 37). Similarly, Correia et al. showed in two surveys carried out in seven African countries (10 years apart) that the prevalence of overweight-obesity was, over time, positively correlated with the socio-economic level of households (6).

Despite the overall poor economy in South Kivu, particularly in urban areas, a part of the population has found informal means to improve their income (7, 21). This new relatively high purchasing power has changed living standards and lifestyles, leading concomitantly to an increasing PFC and a decrease in physical activity. Such reversed tendencies in PFC and physical activity found in this study are observed along the nutritional transition in other African countries (30). Delisle (17) showed that rich households in poor countries, according to their relatively high purchasing power, are exposed to imported processed foods that are rich in added sugar, fat, and, salt, which increase the risk of developing non-communicable diseases and obesity.

The escalating risk of overweight-obesity associated with the enhancement of socio-economic conditions, mediated by a higher PFC, indicates that South Kivu is experiencing an early nutritional transition stage, similar to other sub-Saharan African countries. Our results support the findings of Abrahams et al. (38) showing that African Great Lakes countries neighboring the DRC, particularly the South Kivu region (Rwanda, Burundi, Uganda, Tanzania, and Kenya), are situated at an early phase of the nutritional transition.

In this study, there was no association between overweight-obesity and age, but all individuals with obesity were over 35 years old. This is in line with other African studies demonstrating that age is a risk factor for overweight-obesity (9, 39). As mentioned by Talimula et al. (40) in Benin, physical activity decreases with age and exposes individuals to a sedentary lifestyle, which constitutes a major risk factor for overweight-obesity. Hence, we observed in our study a relationship between age and overweight-obesity mediated negatively by physical activity.

The sociocultural aspects, particularly the valorization of corpulence, also mediate several factors of overweight-obesity during the nutritional transition (15, 16). Indeed, corpulence can be perceived as a sign of peacefulness, wellbeing, good health and social success, as observed in multiple historical social representations. Hence, our study found, both in rural and urban areas, emic (lay) norms of corpulence to be far superior to etic (biomedical) norms. This was also confirmed with dissatisfaction one's body image and the intention to gain weight (41). Although this intention to gain weight could manifest differently, the urban environment can exacerbate this behavior; the weight gain is perceived by less modernized people as a symbol of accession to the urban Eldorado. Therefore, such a sociocultural trend can be a risk factor for overweight-obesity in societies experiencing a rapid nutritional transition, as observed in this study and other African studies (30).

Since women are more at risk of developing overweight and obesity during the early nutritional transition stages (13, 19), studying overweight-obesity in this group from a biocultural perspective helps to better understand this transitional phenomenon in order to propose appropriate public health policies. In addition, our study has benefited from the use of recommended factorial analyses in nutritional epidemiology to assess the relationship between dietary patterns and overweight-obesity (42). Moreover, even with a limited sample, the SEM used in this study enables the identification of the driver pathways between socio-demographic and socio-ecological factors leading to overweight-obesity, a relevant data analysis strategy in line with our previous study conducted in Cameroon (16). However, our study presents some limits, which, after all, do not exclude the value of its results. First, we sampled more elderly subjects in urban areas than in rural areas. This can be explained by the fact that older dwellers choose to migrate to urban areas for finding safety, amidst the continuous insecurity in the region. Contrarily, young rural dwellers might be forced to stay, and for their subsistence, they pursue agricultural work and other activities around natural resource exploitation, such as mining. Then, the cross-sectional design of this study did not allow a longitudinal observation of overweight-obesity and its biocultural determinants. Also, a qualitative approach interpreting deeply their relationships would have been relevant. Future studies should develop such approaches to provide a causal dimension to the relationships observed in this study (30, 43), with a possible gender comparison as well as a national-level survey to obtain an overview of the obesogenic effects of the nutritional transition in the DRC. We also believe that a culturally relevant tool to quantitatively estimate food intake, as developed in Cameroon and Mozambique (44, 45), would provide more arguments to objectively identify the contribution of food consumption in terms of energy intake, and therefore accurately assess its impact on obesity in this region. Finally, in this study, only the BMI was used to assess overweight-obesity. Because this index is a very convenient and appropriate tool to assess the risk factor for morbid obesity at a population level, but not a clinical marker of cardiometabolic diseases at an individual level, our results must be interpreted with caution (46).

In spite of this, this study demonstrated innovatively the relationships between socio-demographic and socio-ecological factors as driver pathways leading to overweight-obesity in the Great Lakes region of the DRC.

## 5 Conclusions

The issue of overweight-obesity among adult women from Bukavu city and its surrounding rural area is a matter of public health concern. We have observed that urban duration, SES, and stoutness valorization are positively associated with overweight-obesity, primarily due to decreased physical activity and elevated frequency of processed food consumption. Understanding the drivers of excess weight, especially in at-risk subgroups, could provide the basis for interventions and public strategies on biocultural trajectories determining overweight-obesity, to prevent its rising prevalence in the Democratic Republic of Congo.

## Data Availability

The main data collected for the study are presented in this article/Supplementary material. Further inquiries can be directed to the corresponding authors.

## References

[1] Simmonet A Chetboun M Poissy J Raverdy V Noulette J Duhamel A. High prevalence of obesity in severe acute respiratory syndrome coronavirus-2 (SARS-CoV-2) requiring invasive mechanical ventilation. Obésity J. (2020) 28:1195–9. 10.1002/oby.22831 32271993 PMC7262326

[2] WHO. Nutrition in the WHO African Region. Brazzaville: World Health Organization. WHO Regional Office for Africa (2017).

[3] Obesity W. World Obesity Atlas 2022. London: Ludgate House Fleet Street. (2022).

[4] Jacobi D Buzelé R Couet C. Peut-on parler de pandémie d'obésité? La Presse Médicale. (2010) 39:902–6. 10.1016/j.lpm.2010.01.014 20663633

[5] Popkin B Adair L Wen Ng S. The nutrition transition to a stage of high obesity and noncommunicable disease prevalence dominated by ultra-processed foods is not inevitable. Obesity Rev. (2022) 23:e13366. 10.1111/obr.13366 34632692 PMC8639733

[6] Correia J Pataky Z Golay A. Comprendre l'obésite en Afrique: poids du développement et des représentations. Revue Méd Suisse. (2014) 10:712–6. 10.53738/REVMED.2014.10.423.0712 24783739

[7] MPSMRM MSP et ICF International. Deuxième enquête démographique et de santé (EDS-RDC II 2013-2014). Rockville, MD: MPSMRM, MSP et ICF International (2014).

[8] Musung JM Muyumba EK Nkulu DN Kakoma PK Mukuku O Kamalo BM. Prévalence du surpoids et de l'obésité chez l'adolescent en milieu scolaire à Lubumbashi, République Démocratique du Congo. Pan African Med J. (2019) 32:11. 10.11604/pamj.2019.32.49.15969 31143354 PMC6522159

[9] Kasereka KO Shongo OL Paluku KL. Déterminants de l'obésité dans la ville de Kinshasa. Int J Progr Sci Technol. (2023) 37:60–8.

[10] Katchunga P Masumbuko BE Lemogoum D Kashongwe ZM Degaute J-P Kabinda JM. Hypertension artérielle chez l'adulte Congolais du Sud Kivu: résultats de l'étude VitaraaHypertension in the adult Congolese population of Southern Kivu: results of the Vitaraa study. La Presse Méd. (2011) 40:315–23. 10.1016/j.lpm.2010.10.036 21376507

[11] Kujirakwinja YB Mulume Oderhwa G Mushengezi Sengey D. Prevalence and determinants of gestationnel Diabetes mellitus in Bukavu, DRC. Ann Africaines Med Univ Kinshasa. (2019) 13:1.

[12] MPMSP. Enquête Démographique et de Santé. République Démocratique du Congo 2007. Calverton, MD: MPMSP et ICF Internationale (2008).

[13] Deslile H. 32 L'obésité est un problème de riches dans les pays en développement. Des idées récues en Santé publique. (2017).

[14] Fidelia AA Emmanuel OT Delali MB. Sociodemographic correlates of obesity among Ghanaian women. Public Health Nutit. (2011) 14:1285–91. 10.1017/S1368980010002879 21029510

[15] Cohen E Gradidge P Ndao A Duboz P Macia E Gueye L. Biocultural determinants of overweight and obesity in the context of nutrition transition in Senegal: a holistic anthropological approach. J Biosoc Sci. (2019) 51:469–90. 10.1017/S0021932018000287 30295213

[16] Cohen E Amougou N Ponty A Guerrien M Wakilongo W Chidumwa G. Direct and indirect determinants of body mass index in both major ethnic groups experiencing the nutritional transition in Cameroon. Int J Environ Res Public Health. (2022) 19:6108. 10.3390/ijerph19106108 35627645 PMC9141336

[17] Delisle H. La transition nutritionnelle, ses déterminants et ses conséquences. In: La Nutrition dans un monde globalisé. Bilan et perspectives à l'heure des ODD. Paris: Marseille, Karthala et IRD (2018). p. 266. 10.4000/books.irdeditions.33914

[18] Ziraba K Fotso JC Ochako R. Overweight and obesity in urban Africa: a problem of the rich or the poor? BMC Public Health. (2009) 9:1–9. 10.1186/1471-2458-9-465 20003478 PMC2803188

[19] Ng M Fleming T Robinson M Thomson B Graetz N Margono C. Global, regional, and national prevalence of overweight and obesity in children and adults during 1980–2013: a systematic analysis for the Global Burden of Disease Study (2013). Lancet. (2014) 384:766–81. 10.1016/S0140-6736(14)60460-8 24880830 PMC4624264

[20] Ministère du Plan-RDC. Monograhie de la province du Sud-Kivu. Kinshasa (2005).

[21] MPBSR-PNUD-RDC. Localisation des Objectifs de développement durable dans le Sud-Kivu. OCDD-RD Congo 2017. Kinshasa (2017).

[22] Dabis F Desenclos J-C. Epidémiologie de terrain Methodes et apllications. JL John Libbey EUROTEXT. (2017).

[23] Kombiané J-F. Habitat et biens d'équipement comme indicateurs de niveau de vie des ménages: bilan méthodologique et application à l'analyse de la relation pauvreté-scolarisation. Etude de la population africaine. (2002) 19:265–83.

[24] Becquey E Savy M Danel P Dabiré HB Tapsoba S Martin-Prével Y. Dietary patterns of adults living in Ouagadougou and their association with overweight. Nutr J. (2010) 9:1–10. 10.1186/1475-2891-9-13 20307296 PMC2848625

[25] Cohen E Bernard JY Ponty A Ndao A Amougou N Saïd-Mohamed R. Development and validation of the body size scale for assessing body weight perception in African populations. PLoS ONE. (2015) 10:1–14. 10.1371/journal.pone.0138983 26536030 PMC4633130

[26] Craig CL Marshall AL Sjöström M Bauman AE Booth ML Ainsworth BE. International physical activity questionnaire: 12-country reliability and validity. Med Sci Sports Exer. (2003) 35:1381–95. 10.1249/01.MSS.0000078924.61453.FB 12900694

[27] Ombeni J Conti MV Cena H Munyuli T. Potential of dried edible caterpillars (lepidoptera: saturniidae) vending on local markets to improve mineral suitability in the diet of the population in Democratic Republic of the Congo. Nat Resour. (2024) 2:10–9. 10.46676/ij-fanres.v5i2.252

[28] Escofier B Pagès J. Analyses factorielles simples et multiples. Objectifs, méthodes et interpretation. Paris: Dunod (2008). p. 328.

[29] Savy M Martin-Prével Y Traissac P Eymard-Duvernay S Delpeuch F. Dietary diversity scores and nutritional status of women change during the seasonal food shortage in rural Burkina Faso. J Nutr. (2006) 136:2625–32. 10.1093/jn/136.10.2625 16988137

[30] Cohen E Amougou N Ponty A Loinger-Beck J Nkuintchua T Monteillet N. Nutrition transition and biocultural determinants of obesity among Cameroonian migrants in urban Cameroon and France. Int J Environ Res Public Health. (2017) 14:696. 10.3390/ijerph14070696 28661463 PMC5551134

[31] Lebailly P Muteba D. Characteristics of urban food insecurity: the case of Kinshasa. Afr Rev Econ Finance. (2011) 3:58–68.

[32] Ambrosini GL Huang R Mori TA Hands BP O'Sullivan T de Klerk NH. Dietary patterns and markers for the metabolic syndrome in Australian adolescents. Nutr Metabol Cardiov Dis. (2010) 20:274–83. 10.1016/j.numecd.2009.03.024 19748245

[33] Debras C Srour B Chazelas E Julia C Kesse-Guyot E Allès B. Ultra-processed foods and health results from the prospective: NutriNet-Santé cohort. Cahiers Nutr Diététique. (2022) 57:222–34. 10.1016/j.cnd.2021.08.004

[34] Sadler CR Grassby T Hart K Raats M Sokolovic M Timotijevic L. Processed food classification: conceptualisation and challenges. Trends Food Sci Technol. (2021) 112:149–62. 10.1016/j.tifs.2021.02.05935284464

[35] WHO. Nutrition in the WHO African Region. Brazzaville: World Health Organization. Brazzaville: WHO Regional Office for Africa (2017).

[36] Ajay IO Adebamowo C Adami H-O Dalal S Diamond MB Bajunirwe F. Urban–rural and geographic differences in overweight and obesity in four sub-Saharan African adult populations: a multi-country cross-sectional study. BMC Public Health. (2016) 16:1–13. 10.1186/s12889-016-3789-z 27793143 PMC5084330

[37] Ginioux C Grousset J Mestari S Ruiz F. Prévalence de l'obésité chez l'enfant et l'adolescent scolarisés en Seine Saint-Denis. SFSP Santé Publique. (2006) 18:389–400. 10.3917/spub.063.0389 17094681

[38] Abrahams Z Mchiza Z Steyn NP. Diet and mortality rates in Sub-Saharan Africa: stages in the nutrition transition. BMC Public Health. (2011) 11:12. 10.1186/1471-2458-11-801 21995618 PMC3209469

[39] Mkuu R Barry A Yonga G Nafukho F Wernz K Gelreath T. Prevalence and factors associated with overweight and obesity in Kenya. Prev Med Rep. (2021) 22:1–7. 10.1016/j.pmedr.2021.101340 34113539 PMC8170142

[40] Talimula D Mizéhoun-Adissoda C Padonou G Aguemon B Barikissou G Ouendo E. Facteurs associés à l'obésité au sein d'un groupe d'usagers du marché Dantokpa (BÉNIN). SFSP Santé Publique. (2019) 31:591–602. 10.3917/spub.194.0591 31959260

[41] Pradeilles R Holdsworth M Olaitan O Irache A Osei-Kwasi HA Ngandu CB. Body size preferences for women and adolescent girls living in Africa: a mixed-methods systematic review. Public Health Nutr. (2022) 25:738–59. 10.1017/S1368980021000768 33593472 PMC9991778

[42] Hu BF. Dietary pattern analysis: a new direction in nutritional epidemiology. Curr Opin Lipidol. (2002) 13:3–9. 10.1097/00041433-200202000-00002 11790957

[43] Shisana O Labadario D Rehle T Simbay L Ramlagan S Zungu N. The South African national health and nutrition examination survey. Cape Town: Online HSRC Press. (2013).

[44] Amougou N Cohen E Mbala M Grosdidier B Bernard J Said-Mohamed R. Development and validation of two food portion photograph books to assess dietary intake among adults and children in Central Africa. Br J Nutr. (2016) 115:895–902. 10.1017/S0007114515005401 26786057

[45] Korkalo L Erkkola M Fidalgo L Nevalainen J Mutanen M. Food photographs in portion size estimation among adolescent Mozambican girls. Public Health Nutr. (2013) 16:1558–64. 10.1017/S1368980012003655 22874096 PMC10271437

[46] Gutin I. Body mass index is just a number: conflating riskiness and unhealthiness in discourse on body size. Sociol Health Illn. (2021) 43:1437–53. 10.1111/1467-9566.13309 34086365 PMC8363552

