# Supplementary material for: Biocultural determinants of overweight-obesity among adult women experiencing the nutritional transition in the Democratic Republic of Congo
Source: Front Nutr. 2024 Nov 25;11:1341710. doi: 10.3389/fnut.2024.1341710 (PMC11625540; doi:10.3389/fnut.2024.1341710)
Supplement: Supplementary file 1 [file Data_Sheet_1.PDF]

APPENDIX : SUPPLEMENTARY MATERIAL

APPENDIX 1: Flow chart diagram of the study participants

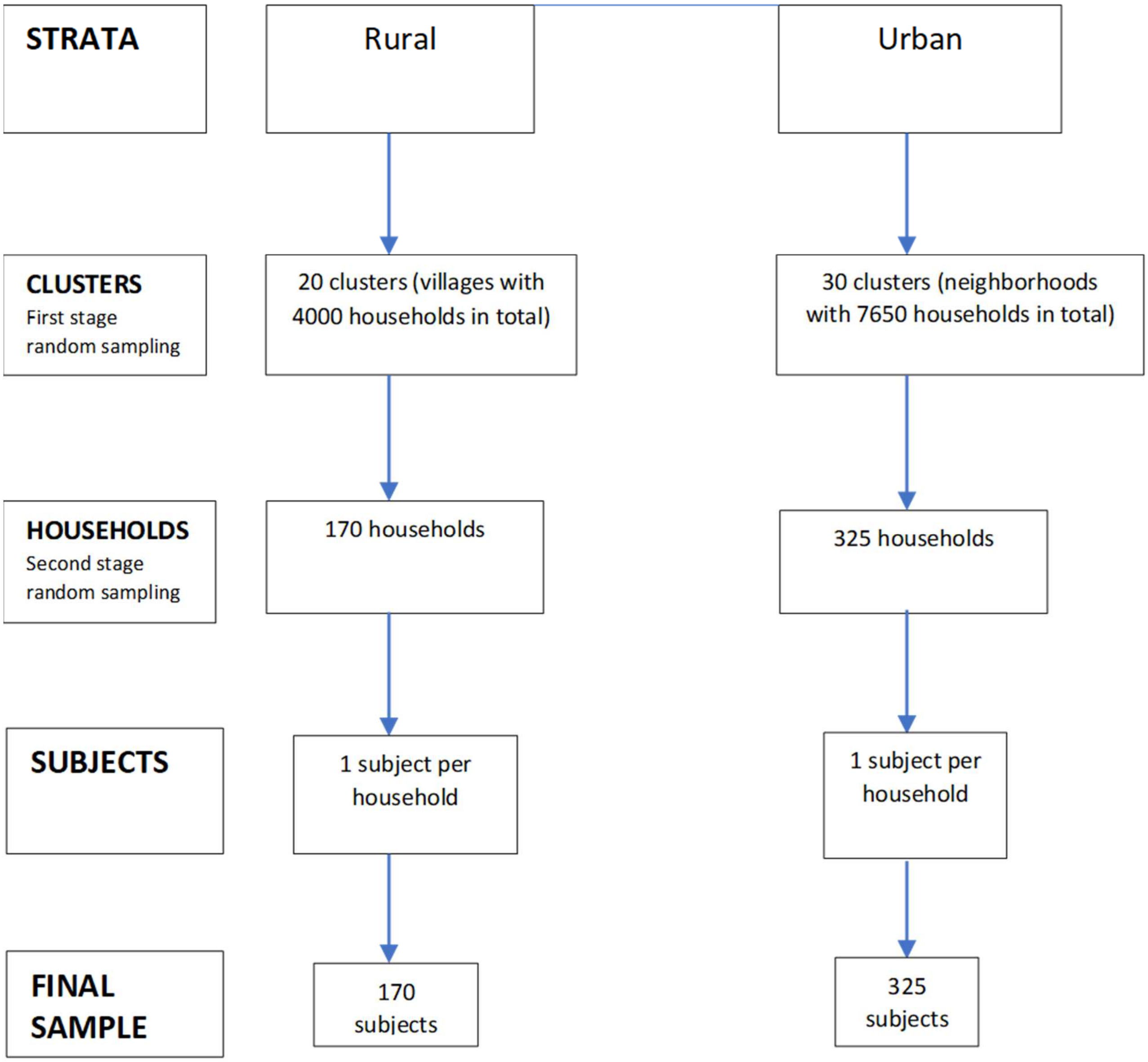

Figure S1: Flow chart diagram of study participants

APPENDIX 2 : Methods results of socio-economic scores

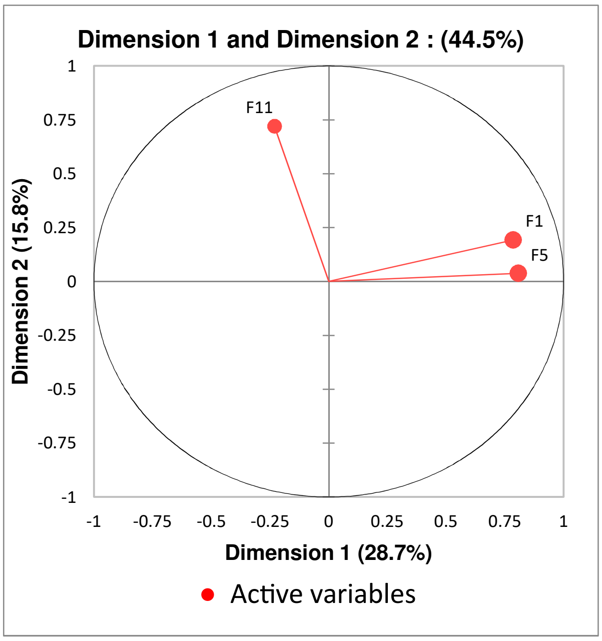

Figure S2. PCA: Correlation circle of owned household goods. The displayed factors synthesize different type of assets, and discriminate the most Dimensions 1 and 2.

APPENDIX 3 : Method results of food consumption scores

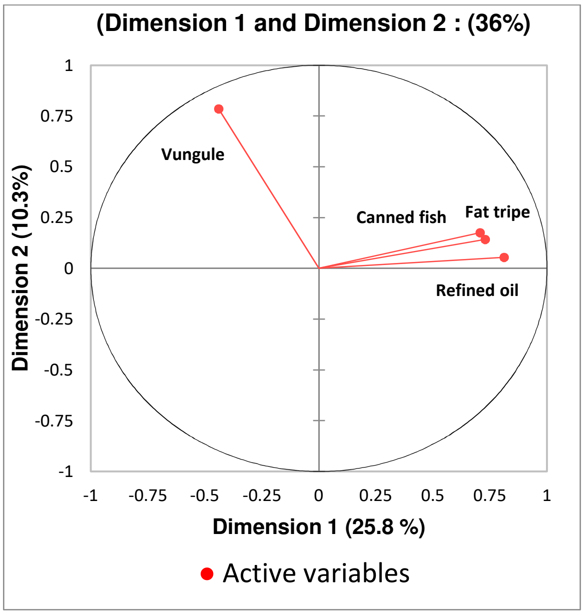

Figure S3. PCA: Correlation circle of food consumption variables. The food variables included discriminate the most Dimensions 1 and 2.

Table S1. Correlations between variables and factors after Varimax rotation

	F1	F2
Cake	**0.541**	-0.175
Semolina	**0.650**	-0.095
Refined oil	**0.781**	-0.228
Polished rice	**0.588**	-0.023
Soda-juce	**0.500**	-0.143
Canned fish	**0.723**	-0.077
Fofou	-0.090	**0.648**
Palm sauce	0.042	**0.647**
Fry sauce	-0.214	**0.508**
Fatty meat	**0.503**	-0.122
Bean	-0.059	0.435
Nido (Powdered milk)	0.434	0.045
Beer	**0.522**	0.018
Sugar	**0,662**	-0.092
Break fast	**0.611**	-0.068
Tuber	-0.201	**0.597**
Snack freq	**0.633**	-0.137
Fat tripe	**0.733**	-0.117
Vungule	-0.144	**0.889**
Green leg	**0.423**	0.009
Meat	**0.382**	0.011
Palm oil	**-0.376**	0.019
Pondou	0.112	**0.407**
